# Epithelial Cell-Derived Extracellular Vesicles Trigger the Differentiation of Two Epithelial Cell Lines

**DOI:** 10.3390/ijms23031718

**Published:** 2022-02-02

**Authors:** Tiago Ramos, Mohit Parekh, Stephen B. Kaye, Sajjad Ahmad

**Affiliations:** 1 Department of Eye and Vision Science, University of Liverpool, Liverpool L7 8TX, UK; ramostiago254@gmail.com (T.R.); s.b.kaye@liverpool.ac.uk (S.B.K.); 2 Institute of Ophthalmology, Faculty of Brain Sciences, University College London, London EC1V 9EL, UK; m.parekh@ucl.ac.uk; 3 St Paul’s Eye Unit, Royal Liverpool University Hospital, Liverpool L7 8XP, UK; 4 Moorfields Eye Hospital NHS Foundation Trust, London EC1V 2PD, UK

**Keywords:** cell differentiation, conjunctiva, cornea, epithelial cell, extracellular vesicles, exosomes, miRNA, ocular surface

## Abstract

Extracellular vesicles (EVs), specifically exosomes, carry a cell-type dependent cargo that is transported to the recipient cell and translated in the presence of a required machinery. Differences in the cargo carried by the corneal and conjunctival-derived EVs could be the agent that triggers the transdifferentiation of these two cell populations. Therefore, this study investigates the role of EVs in triggering the plasticity of corneal and conjunctival epithelial cells and identifies prospective miRNA and genes responsible for maintaining ocular surface homeostasis. The EVs were extracted from the conditioned media (after starving) of corneal epithelial (hTCEpi) and conjunctival (HCjE-Gi) cell lines using ultracentrifugation. HCjE-Gi cells were cultured with hTCEpi-derived EVs and vice-versa. The EVs were characterized as exosomes using Nanosight and Flow cytometry. KRT3 and KRT12 were used as associated corneal markers, whereas KRT7 and KRT13 were used as associated conjunctival markers with ΔNp63 as a differentiation marker. Shift of these markers was an indication of transdifferentiation. The cargo of the extracted exosomes from both the cell types was explored using next-generation sequencing. The hTCEpi-derived EVs induced conjunctival epithelial cells to express the corneal-associated markers KRT3 and KRT12, losing their conjunctival phenotype at both the mRNA and protein level. Simultaneously, HCjE-Gi-derived EVs induced corneal epithelial cells to express the conjunctival associated markers KRT7 and KRT13, losing their corneal phenotype. This process of differentiation was accompanied by an intermediate step of cell de-differentiation showed by up-regulation in the expression of epithelial stem cell marker ΔNp63, also shown on the ex vivo human cadaveric donor corneas. miRNA molecules (total of 11 including precursor and mature) with significant differences in their relative abundance between the two populations (*p* < 0.05) were found and investigated. miR-9-5p expression was higher in HCjE-Gi cells and HCjE-Gi-derived EVs when compared to hTCEpi cells and hTCEPi-derived EVs (*p* < 0.001). The results suggest that EVs released by the two cell types have the ability to influence the transdifferentiation of human conjunctival and corneal epithelial cells. miR-9-5p could have a role in stem cell homeostasis and cell differentiation via *HES-1* gene.

## 1. Introduction

**Figure 1:**
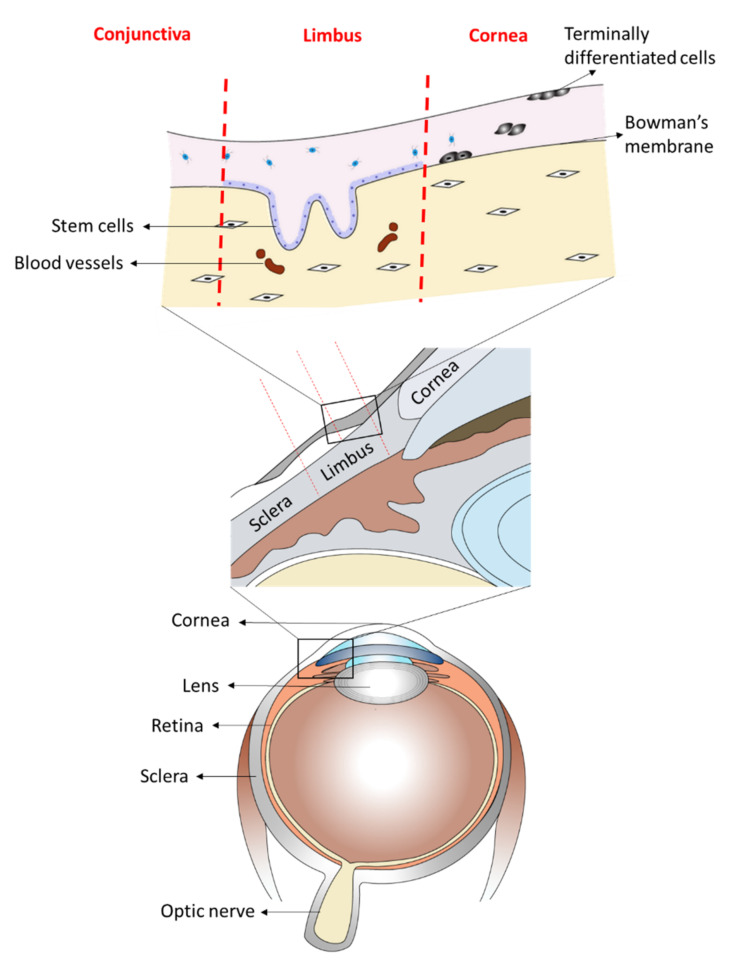
A scientific illustration of human ocular anatomy showing the anterior part of the eye with two phenotypically and functionally distinct structures. The transparent front layer of the eye, the cornea, which is covered by the corneal epithelium and the anterior sclera, that encircles the cornea and covered by the conjunctival epithelium that also extends to cover the inner surface of the eyelids. Both these cell types are separated by the limbus, which is the reservoir of the stem cells for the corneal epithelium.

The anterior part of the eye is composed of two phenotypical and functionally distinct structures: the cornea and the conjunctiva. The cornea is the clear front of the eye and is covered by the corneal epithelium. The anterior sclera, which encircles the cornea, is covered by the conjunctival epithelium that also extends to cover the inner surface of the eyelids. Both epithelia are separated at the peripheral edge of the cornea by a narrow band of tissue known as the limbus, which also harbors the stem cells for the corneal epithelium, known as the limbal stem cells [1,2,3,4] (Figure 1). Following limbal stem cell deficiency (LSCD), conjunctival epithelial cells may migrate onto the cornea, a process called conjunctivalisation [5]. This results in loss of corneal clarity and visual impairment [5,6,7,8,9,10]. Reports have suggested that corneal conjunctivalisation is usually followed by the transdifferentiation of the migrating conjunctival epithelial cells towards a corneal epithelial-like phenotype, a mechanism still not fully understood [5,6,11]. Recently, it has been shown that the process of conjunctival transdifferentiation is incomplete and the newly regenerated epithelium fails to express the corneal specific marker, keratin (KRT) 12 [12]. Kurpakus et al., however, showed that conjunctival epithelial cells cultured on top of intact corneal epithelial basement membrane will express KRT 12 [10], suggesting that with the appropriate cues, the process of conjunctival transdifferentiation towards a corneal epithelial phenotype can be induced. Many agents have been shown to trigger the process of transdifferentiation both in vitro and in vivo; some of these include transcription factors [13,14,15] and proteins from the extracellular matrix [10].

Corneal and conjunctival epithelial cells belong to two phenotypically different lineages [16]. They express different keratins [17,18,19,20,21] and have differing cell-cell and cell-matrix adhesions. KRTs are intermediate filaments (type I and type II) that aid in forming the cytoskeleton of epithelial cells, providing them with structural integrity. KRT3 and particularly KRT12 form the most widely accepted dimer for corneal epithelial cells [17,18,19], whereas KRT7 and KRT13 have been accepted for the conjunctival epithelial cells [17,20,21]. Functionally, as compared to the corneal epithelium, which is clearer and more tightly adherent, the conjunctival epithelium is hazier, has fewer tight junctions, and is less adherent to its basement membrane complex [22,23]. The possibility of corneal and conjunctival epithelial transdifferentiation has been studied in disease resulting in loss of corneal clarity by conjunctivalisation and hence blindness [5,6,7,8,9,10]. Although being suggested as the result of environmental modulation [10], the precise mechanisms of conjunctival transdifferentiation are not still fully understood.

Extracellular vesicle (EV) trafficking is one of the proposed mechanisms for intercellular communication in multicellular organisms [24]. Produced by different mechanisms with different subcellular origins and categorized by size, three types of EVs have been identified; the apoptotic bodies (1 to 5 µm in diameter), the microvesicles (up to 1µm in diameter) and exosomes (40 to 150 nm in diameter) [25,26]. Exosomes are intraluminal membrane vesicles of endocytic origin that form from the inward budding of the endosomal membrane [27,28,29,30]. They contain mRNA and micro-RNA (miRNA) molecules, small amounts of DNA, and proteins such as transcription factors, cytokines and growth factors [31]. By transferring their cargo between the producing and the recipient cell (via processes that are not completely understood), exosomes and other EVs have been shown to play important roles in cell differentiation [32], proliferation [33] and therefore provide a potential mechanism for triggering cell transdifferentiation.

This study, therefore, investigates whether EVs derived from the corneal epithelial cell type induce plasticity in the conjunctival epithelial cells and vice versa, particularly in terms of keratin profiling shift at both mRNA and protein levels. Furthermore, we also document differences in exosomal cargo derived from conjunctival and corneal epithelial cells and identify the distinct miRNA players influencing cell differentiation of the two studied cell types.

## 2. Results

### 2.1. Isolation, Characterization and Uptake of Corneal and Conjunctival Epithelial Cells-Derived Extracellular Vesicles

**Figure 2:**
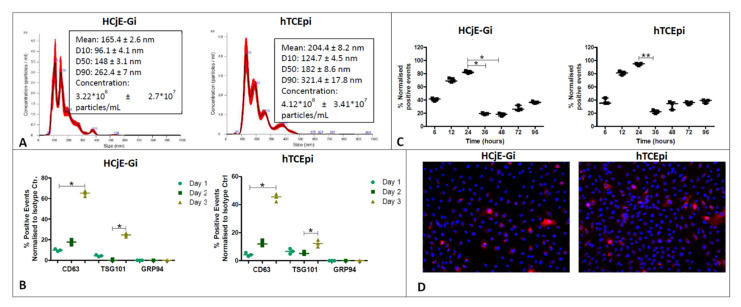
Characterization and internalization of the EVs from both the cell types. (**A**) Size distribution and particle concentration of the extracted EVs from conditioned medium obtained after culturing the cells for three days as assessed by NanoSight. The percentage undersize is indicated by D10, D50, and D90. (**B**) Characterization using flow cytometry analysis showing the percentage of positive events that are normalized against its isotype control. (**C**) Internalization and/or docking profiles of XenoLight DiR labelled exosomes of hTCEpi cell-derived exosomes by HCjE-Gi cells and HCjE-Gi cell-derived exosomes by hTCEpi cells. Results are normalized against non-labelled exosomes (Data is represented as median ± range, *n* = 3, Kruskal–Wallis test followed by Dunn’s Multiple Comparison test, * *p* < 0.05, ** *p* < 0.01). (**D**) The presence of exosomes in the samples was observed following the DiL labeling on HCjE-Gi and hTCEpi cells. Dil labelled exosomes—red; nuclei stained with DAPI—blue.

NanoSight^®^ analysis confirmed the presence of EVs in both HCjE-Gi (conjunctival) and hTCEpi (corneal) conditioned media, at concentrations of 4.12 × 10^8^ and 3.22 × 10^8^ particles/mL, respectively (Figure 2A). The modal peak showed values within the size range for exosomes that is 112 nm for HCjE-Gi (conjunctival) cells and 133.3 nm for hTCEpi cells (corneal) [25,26]; however, smaller peaks around 190 nm and 150 nm were also observed. In accordance with guidelines from the International Society of Extracellular Vesicles, exosomes can be identified by the following positive markers: CD63, a membrane-bound tetraspanin, and TSG101, an endosome binding protein; and the negative marker GRP94, an endoplasmic reticulum protein [34]. The absence of EVs in fresh BPE-free culture medium (negative control) was confirmed by flow cytometry. Flow cytometry showed the presence of CD63- and TSG101-positive events and the absence of GRP94-positive events in EVs extracted from both HCjE-Gi (conjunctival) and hTCEpi (corneal) conditioned media (Figure 2B) [34], thus suggesting that majority of the isolated EVs showed exosome-associated features. The percentage of DiR-labelled hTCEpi-derived and HCjE-Gi-derived exosomes internalized and/or docked to the cell membrane of HCjE-Gi and hTCEpi cells respectively, which increased over-time. Flow cytometry showed that the percentage of fluorescing HCjE-Gi and hTCEpi-derived EVs increased within the first 24 h of culture (*p* ≤ 0.05) and diminished to baseline levels thereafter (Figure 2C). The cellular uptake of the EVs was also observed at 24 h following DiL labelling (Figure 2D).

### 2.2. HCjE-Gi Cells Cultured with hTCEpi-Derived EVs Lose Conjunctival Epithelial Marker Expression and Gain Corneal Epithelial Marker Expression and hTCEpi Cells Cultured in HCjE-Gi-Derived EVs Lose Corneal Epithelial Marker Expression and Gain Conjunctival Epithelial Marker Expression

HCjE-Gi cells were cultured in the presence of hTCEpi-derived EVs and examined for the expression of well-recognized terminally differential corneal epithelial cell markers KRT3 and KRT12 [18,19], terminally differentiated conjunctival epithelial cell markers KRT7 and KRT13 [17,20,21], limbal stem cell marker ABCB5 and the epithelial stem cell marker ΔNp63 [35,36]. As a control, HCjE-Gi cells were also cultured in the presence of HCjE-Gi-derived EVs. Similar to the HCjE-Gi cells, hTCEpi cells were cultured in the presence of HCjE-Gi-derived EVs and analyzed for the same markers. As a control, hTCEpi cells were also cultured in medium containing hTCEpi-derived EVs.

#### 2.2.1. Real-Time PCR

**Figure 3:**
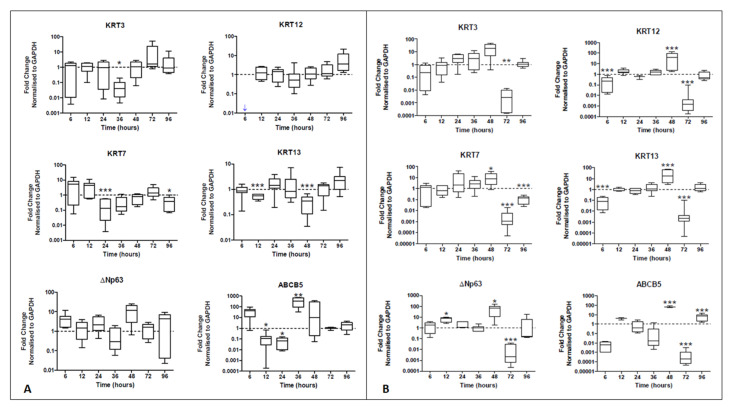
Real-time qPCR data showing the expression of epithelial cell markers by (**A**) HCjE-Gi and (**B**) hTCEpi cells when cultured in the medium containing hTCEpi-derived and HCjE-Gi-derived exosomes compared to the cells cultured in medium containing HCjE-Gi-derived and hTCEpi-derived exosomes, respectively. The data were collected and assessed over 96 h time period. (Data is represented as median ± 5–95 percentile, *n* ≥ 6, Mann–Whitney test, Bonferroni-corrected *p*-value, * *p* < 0.05, ** *p* < 0.01, *** *p* < 0.001). Dashed line represents the basal expression of the markers of interest when cells are cultured on their own respective exosomes-containing medium. Abbreviations used GAPDH: glyceraldehyde 3-phosphate dehydrogenase, KRT: keratin, ABCB5: ATP-binding cassette sub-family B member 5.

Real-time PCR data showed a decrease in KRT7 expression at 24 h (7.7-fold decrease, *p* < 0.001) and then again at 96 h (7.7-fold decrease, *p* < 0.05). The expression of KRT13, another conjunctival epithelial marker, also decreased at 12 h and then again at 48 h (1.8- and 2.8-fold decrease, both *p* < 0.001). Regarding the expression of corneal epithelial cell markers, although there was no statistically significant increase (*p* > 0.05), from 36 h onwards, a steady increase in both KRT3 and KRT12 expression was observed. Although several peaks in ΔNp63 expression were appreciated at 6 h and 48 h when hTCEpi cells derived extracellular vesicles were used (4.1- and 12-fold increase), no statistically significant differences in ΔNp63 expression were noted (*p* > 0.05) (Figure 3A). The expression of ABCB5 peaked at 36 h, being significantly increased (*p* < 0.01) when hTCEpi-derived extracellular vesicles were used and compared to HCjE-Gi-derived extracellular vesicles (329-fold increase).

Real-time PCR data showed a reduction in the expression of KRT12 at 6 h (10-fold decrease, *p* < 0.001). Importantly, there was an increase in the expression of conjunctival markers KRT7 (8.1-fold increase, *p* < 0.05) and KRT13 (17-fold increase, *p* < 0.001) at 48 h. This corresponded to an increase in the expression of ΔNp63 also at 12 h and 48 h (respectively 7.8- and 66-fold increase, *p* < 0.05). Similar results were observed in the expression of ABCB5. A decline in the expression of all markers was observed at 72 h (all at least *p* < 0.01) (Figure 3B).

#### 2.2.2. Flow Cytometry

**Figure 4:**
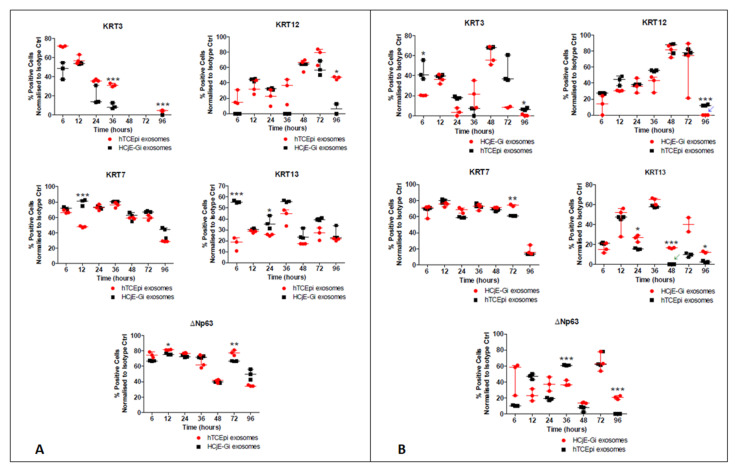
Flow cytometry analysis showing the expression of epithelial cell markers by (**A**) HCjE-Gi and (**B**) hTCEpi cells when cultured in the medium containing hTCEpi cells- or HCjE-Gi cells-derived exosomes, respectively. The results were compared to the cells cultured in the medium containing HCJE-Gi cells or hTCEpi cells-derived exosomes. The data were collected and assessed over 96-h time period. The percentage of positive events normalized against isotype control is shown. (Data is represented as median ± interquartile range, *n* ≥ 3, Mann-Whitney test, Bonferroni corrected *p*-value, * *p* < 0.05, ** *p* < 0.01, *** *p* < 0.001). Abbreviations used Ctrl: control, KRT: keratin.

Flow cytometry data showed a decrease in the percentage of cells expressing the conjunctival markers KRT7 at 12 h (*p* < 0.001) and KRT13 (*p* < 0.001) at 6 h to only about 20% of the HCjE-Gi cells expressing the latter. Regarding corneal epithelial cell markers, there was an increase in the percentage of cells expressing KRT3 at 36 h and 96 h (*p* < 0.001) and KRT12 (*p* < 0.05) at 96 h. There was also an increase in the proportion of cells expressing ΔNp63 at 12 h (*p* < 0.05) and at 72 h (*p* < 0.01) (Figure 4A). The flow cytometry data showed a reduction in the percentage of hTCEpi cells expressing KRT3 at 6 h and 96 h (*p* < 0.05) and KRT12 at 96 h (*p* < 0.001). Regarding conjunctival epithelial cell markers, there was a higher percentage of cells expressing KRT7 at 72 h (*p* < 0.01) and KRT13 at 24 h, 48 h, and 96 h (at least *p* < 0.05). These epithelial cell marker changes were associated with significant changes in the proportion of cells expressing ΔNp63: a decrease at 36 h and an increase at 96 h (both *p* < 0.001) (Figure 4B).

Real-time PCR and the flow cytometry data showed a loss in expression of conjunctival epithelial cell markers when the HCjE-Gi (conjunctival) cells were cultured in the presence of hTCEpi-derived (corneal) EVs compared to HCjE-Gi cells cultured in a medium containing their own EVs, and a definite loss of corneal epithelial cell markers associated with an increase in conjunctival epithelial cell markers when the hTCEpi (corneal) cells were cultured in medium containing HCjE-Gi-derived (conjunctival) EVs. Certainly, at the protein level, there is also an increase in corneal epithelial cell markers.

### 2.3. Distinct Small RNA Profile of HCjE-Gi and hTCEpi-Derived Extracellular Vesicles

HCjE-Gi and hTCEpi-derived extracellular vesicles showed mRNAs encoding for all the proteins assessed, with the exception of KRT12 and KRT13.

Both, precursor and mature miRNA molecules totaling 1668 were detected in the analyzed sample, out of which 337 were only specific to HCjE-Gi-derived EVs and 282 to hTCEpi-derived EVs. However, 1049 miRNA molecules were found in both. Eleven miRNA molecules (precursor and mature) with significant differences in their relative abundance between the two populations (*p* < 0.05 and log2foldchange > 2) are shown in Table 1.

### 2.4. miR-9-5p Expression Profile on HCjE-Gi, hTCEpi Cells and Their Derived Extracellular Vesicles

**Figure 5:**
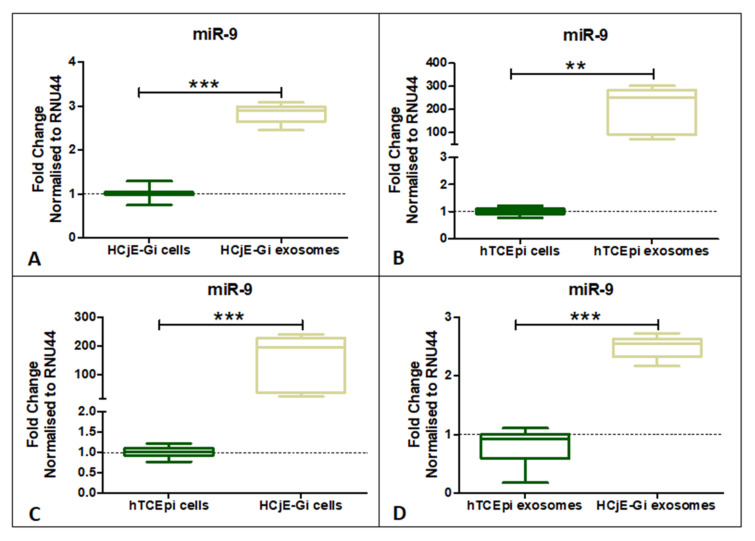
Expression profile of miR-9-5p on HCjE-Gi and hTCEpi cells and their derived extracellular vesicles. miR-9-5p showing significantly higher expression in (**A**) HCjE-Gi and (**B**) hTCEpi cell-derived exosomes. (**C**) miR-9-5p showing higher expression in HCjE-Gi cells compared to hTCEpi cells. (**D**) miR-9-5p expressed more in HCjE-Gi exosomes compared to hTCEpi exosomes. (Data is represented as median ± 5–95 percentile, *n* ≥ 3, Mann–Whitney test, Bonferroni-corrected *p*-value, ** *p* < 0.01, *** *p* < 0.001).

miR-9-5p expression was investigated in both the cell lines and their EVs. miR-9-5p showed significantly higher expression in HCjE-Gi and hTCEpi-derived EVs when compared to the cell of origin (Figure 5A,B) (*p* < 0.01). Additionally, miR-9-5p expression was found to be significantly higher in HCjE-Gi cells and HCjE-Gi-derived EVs when compared to hTCEpi cells and hTCEpi-derived EVs, respectively (Figure 5C,D) (*p* < 0.001).

### 2.5. Expression of Corneal and Conjunctival Biomarkers and HES-1 on Corneal Tissues Following Treatment with HCjE-Gi and hTCEpi-Derived Extracellular Vesicles in an Ex Vivo Study

**Figure 6:**
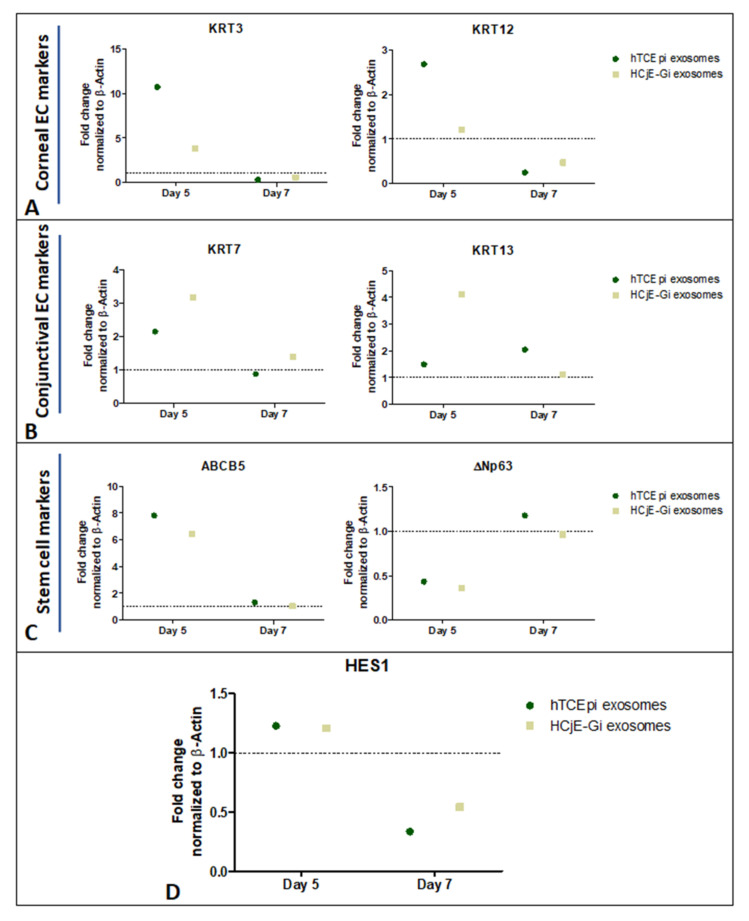
Ex vivo study on human cadaveric donor tissues following treatment with HCjE-Gi and hTCEpi cell-derived EVs. Overexpression of (**A**) KRT3 and KRT12 at day 5, (**B**) KRT7 and KRT13 at day 5 and day 7 and (**C**) ABCB5 at day 5 and ΔNp63 at day 7 following treatment with only hTCEpi cell-derived EVs. (**D**) HES-1 was overexpressed at day 5. (*n* = 1 for each time point).

Corneal epithelial cell markers KRT3 and KRT12 were higher at day 5 (Figure 6A), whereas the conjunctival markers showed higher expression at both day 5 and day 7 when the tissues were treated with HCjE-Gi and hTCEpi-derived EVs (Figure 6B). Stem cell marker ABCB5 showed a higher expression at day 5 but not at day 7, whereas ΔNp63 only showed a higher expression at day 7 when the tissues were treated with hTCEpi-derived EVs (Figure 6C). Moreover, HES-1 was expressed at day 5 on the tissues when treated with both, HCjE-Gi and hTCEpi-derived EVs (Figure 6D).

## 3. Discussion

Over the past few years, the use of EVs as therapeutic agents has increased dramatically as EVs constitute an important mechanism for the transfer of bioactive molecules between different cells both in physiological and pathological conditions [37,38,39]. However, very few studies have reported their roles in eye and vision science [32,40,41].

Several ocular diseases involve the loss of cells from the ocular surface. Stem cell-based therapies have therefore been successfully used to restore the function of the impaired structures [42]. Recently, exosomes derived from different stem cell types have shown to be involved in a wide panoply of therapeutic functions. Exosomes are enriched in major histocompatibility complexes and do not respond to immunosuppressive molecules [43,44,45,46], prompting their potential use as therapeutic agents. Intravitreal injections of mesenchymal stem cells (MSC)-derived exosomes have been used to support retinal ganglion cells in a glaucoma model, preventing axonal loss and degeneration following injury [47]. MSC-derived exosomes have also been shown to possess anti-inflammatory properties that may be applicable to inflammatory eye diseases [48,49].

The two types of epithelial cells of the ocular surface studied here are adjacent to each other in vivo but clearly demarcated in healthy conditions. They both have access to the tear film that may serve as a means of communication, possibly via EVs. To date, tear fluid as a source of exosomes has not been extensively studied. The presence of exosomes, containing RNA and DNA molecules in tears of healthy human individuals has already been shown [50]. The corneal and conjunctival epithelial cells are phenotypically different with differences in their keratin profiles [17,18,19,20], their basement membrane composition [51] and their adhesion complexes [22,23]. The study described here shows that the two different epithelial cells of the ocular surface release EVs that possess exosome-like properties, playing a role in changing keratin expression towards the epithelial cell type of EV origin and therefore enabling plasticity of these two terminally differentiated cell types. Corneal epithelial cell-derived EVs, and exosomes particularly, may therefore play a role in modifying the corneal epithelium following disease where the corneal surface is conjunctivalised. Studies in the late 1980s and 1990s suggested that conjunctival epithelial transdifferentiation was possible in vivo in rabbit models when conjunctival cells were cultured on a corneal substrate with an intact basement membrane [52,53,54,55], contradicting previous studies that report incomplete transdifferentiation towards the corneal epithelium [7,8,9,56].

Despite being out of the range size of any other EVs rather than exosome category, peaks at larger diameter sizes were observed due to the absence of a perfect focus inherent to NanoSight ^®^ (NanoSight NS300 instrument, Amesbury, UK) technique. An uptake of EVs at 24 h (DiL label) and the decrease in XenoLight DiR-positive events after 24 h suggested that labelled EVs docked and/or were taken up by both types of epithelial cells; however, their membrane degraded, and their cargo eventually released and metabolized by the cells. Recent studies have revealed that exosomes affect a wide range of biological processes such as cell differentiation, migration, and proliferation, through the cargo transfer from the originating cell to the recipient cell [41,57,58]. Real-time PCR and flow cytometry studies showed that there was a reduction in the keratin profile of the original cell type and, certainly in the case of hTCEpi (corneal) cells, an increase in the keratin profile of the cell type from which the EVs were obtained. The study also suggested that this process involves an intermediate step of cell de-differentiation, as increased expression levels of the epithelial stem cell marker ΔNp63 were observed. A similar trend was also observed in the ex vivo model.

EVs, and particularly exosomes, carry a cell-type dependent cargo that is transferred to the recipient cell and translated in the presence of the necessary machinery [37]. Differences in the cargo carried by the corneal and conjunctival-derived EVs may be the agent that triggers the transdifferentiation of these two cell populations.

In our study, we assessed the presence of mRNA molecules that encode for KRTs that distinguish corneal and conjunctival epithelial cells. Both extracellular vesicles populations showed to contain the mRNA to encode KRT3, KRT7, and ΔNp63. These data and the notion that exosomal mRNA can be translated [37] further aid in understanding the rapid cell response in terms of protein turn-over.

In all types of EVs, including exosomes, miRNAs have been found in large amounts, which have various effects in the recipient cell due to their roles in modulating several biological processes, including cell differentiation. The comparative exosomal miRNA profiling between the two EVs population helps to reveal the mechanism of exosomal function, particularly in cell differentiation. miR-598 and miR-34c, significantly highly expressed in hTCEpi-derived EVs, have shown to be downregulated in cancer tissues and to stimulate epithelial–mesenchymal transition [59], regulate embryonic stem cell [60] and promote osteoblast differentiation [61]. miR-146 and miR-155, have shown to be highly expressed in HCjE-Gi-derived EVs and are overexpressed in limbus versus central cornea, suggesting its possible role in LSCs homeostasis [62] and antagonize transcription factors that regulate cell differentiation [60,63].

Paradoxically, miR-9 was found to be significantly expressed in HCjE-Gi-derived EVs and has been shown to suppress the expression of the transcription factor HES-1, inducing neural stem cell differentiation [64]. HES-1 is mainly expressed in corneal progenitor cells co-localized with ΔNp63 at the limbal region and its forced expression is linked with epithelial stem cell proliferation and maintenance, therefore inhibiting their differentiation [65]. Significant differences in content from the two populations of the EVs are mainly related to miRNA molecules involved in maintaining and/or promoting an undifferentiated cell state. This is highly expressed in EVs derived from HCjE-Gi cells, with the exception of miR-9. Because of their role in antagonizing HES-1, miR-9 expression was further investigated on both cell lines and their EVs. The results showed that miR-9 is enriched in both HCjE-Gi cells and their derived EVs when compared to hTCEpi cells and hTCEpi-derived EVs, respectively. Its upregulation in HCjE-Gi cells and derived EVs suggest that these have higher potential to antagonize HES-1 and therefore promote cell differentiation. In accordance with the published literature, these data suggest that miRNAs are selectively incorporated into EVs being their miRNA content different from their cell of origin [66,67,68].

On the ex vivo tissues, the epithelial and conjunctival epithelial cell markers were observed following the treatment of the tissues with epithelial and conjunctival cell-derived EVs. However, specifically, ΔNp63 was overexpressed only when the tissues were treated with hTCEpi-derived EVs. This could further imply the transdifferentiation potential, specificity and communication of specific cell-derived EVs in maintaining stem cells. It has been found that HES-1 is mainly expressed in the corneal epithelial stem/progenitor cells and is responsible for regulating corneal development and homeostatic function. It is not found in the differentiated corneal epithelial cells [65]; however, although a pilot study, expression of HES-1 following both the HCjE-Gi and hTCEpi cell-derived exosome treatment was observed on day 5 on the tissues. This further highlights that EVs from the corneal and conjunctival epithelial cells possessing miR-9-5p can modulate HES-1 and can play an important role in maintaining the ocular surface homeostasis. The specificity of EVs in increased expression of ΔNp63 following treatment with hTCEpi cell-derived EVs and the expression of HES-1 for ocular surface maintenance requires further evaluation for a potential therapeutic treatment approach.

## 4. Materials and Methods

### 4.1. Ethical Statement

The study was approved by the University of Liverpool IRB. For ex vivo analysis, the tissues that were unsuitable for transplantation due to poor endothelial cell count were obtained from The Veneto Eye Bank Foundation, Venice, Italy following a written consent from the donor’s next of kin to be used for research.

### 4.2. Cell Culture

HCjE-Gi, a conjunctival epithelial cell line [69] was cultured in keratinocyte serum-free medium (KSFM) (Gibco ™ ThermoFisher Scientific, Waltham, MA, USA) supplemented with 0.2% Bovine Pituitary Extract (BPE), 0.2 ng/mL of epidermal growth factor (EGF) (all supplied with the medium), 1% penicillin/streptomycin (Sigma-Aldrich, Darmstadt, Germany), and 0.4 mM of CaCl_2_ (Sigma-Aldrich, Darmstadt, Germany).

hTCEpi, a corneal epithelial cell line [70] was cultured in KSFM (Gibco ™ ThermoFisher Scientific, Waltham, MA, USA) supplemented with 0.2% BPE, 0.23 ng/mL EGF (all supplied with the medium), and CaCl_2_ to a final concentration of 0.13 mM.

### 4.3. Extraction, Quantification and Sizing, Characterisation and Cellular Uptake of the EVs

#### 4.3.1. Extraction Using Ultracentrifugation

The cells were cultured in BPE- and exosome-free KSFM for 72 h upon 80% confluency. The conditioned medium was collected and centrifuged at 500× *g* (Centrifuge 5417, Eppendorf, Hamburg, Germany) for 5 min at 4 °C to remove any dead cells. The supernatant was collected and re-centrifuged at 2000× *g* for 10 min at 4 °C to remove any remnants from the cell. The following supernatant was then filtered using a 0.22 µm filter (Merck Millipore, Burlington, MA, USA), ensuring only small molecules passed through the filter. Conditioned medium was collected (approximately 32 mL) in OptiSeal™ tubes (Beckman Coulter, Brea, CA, USA) and ultracentrifuged at 100,000× *g* (90Ti fixed angle rotor, Beckman Coulter, Brea, CA, USA) for 2 h at 4 °C. The pellet obtained following ultracentrifugation was re-suspended in sterile phosphate-buffered saline (PBS, Gibco™ ThermoFisher Scientific, Waltham, MA, USA) to wash out any potential media remains. The suspension was re-centrifuged with the same specifications and the resulting pellet was re-suspended in 200 µL of PBS. The obtained EV sample in PBS was stored at −80 °C for further experiments.

#### 4.3.2. Quantification and Sizing by NanoSight

Approximately 5 µL of the EV suspension was diluted in PBS (1:150) and the resulting suspension was used for NanoSight^®^ analysis, which also included quantification and sizing. The experiment and the reading were followed as per the manufacturer’s instructions (NanoSight NS300 instrument, Amesbury, UK). The specifications included maintaining a constant temperature at 22 °C and viscosity of the water at 0.953cP. For analysis, 5 repeats at 1498 frames were collected at a rate of 25 fps.

#### 4.3.3. Characterization by Flow Cytometry

The suspension containing EVs was mixed well and incubated with 10 µL of aldehyde/sulphate latex beads (ThermoFisher Scientific, Waltham, MA, USA) for 15 min at room temperature (RT). A final volume of 1 mL was prepared by adding PBS to the solution and incubated overnight at 4 °C on a bench test tube rotator wheel at 20 rpm (Stuart ^®^ Equipment, Staffordshire, ST15 OSA, UK). Glycine (Sigma-Aldrich, Darmstadt, Germany) was added to the solution at a final concentration of 100 mM and incubated at RT for 30 min. The resulting solution was centrifuged (all the centrifugation steps were performed under RT at 4000 rpm for 3 min). Following the centrifugation step, the pellet was washed thrice in 1 mL of 0.5% bovine serum albumin (BSA, Sigma-Aldrich, Darmstadt, Germany) diluted in PBS. The pellet was re-suspended in 100µL of primary antibody (Supplementary Materials Table S1) diluted in buffer containing 0.5% BSA in PBS and incubated in the dark for 30 min at 4 °C. After washing and re-centrifugating, the resulting pellet was re-suspended in 100 µL of appropriate secondary antibody (Supplementary Materials Table S2) diluted in the same buffer. The suspension was incubated in the dark for 30 min at 4 °C. The resulting pellet following washing and centrifugation was re-suspended in 500 µL of the same buffer. The final suspension was analyzed using BD Accuri C6 flow cytometer (Laser 488 nm, filter 533/30, BD Biosciences, Franklin Lakes, NJ, USA) and the results were analyzed using a BD Accuri C6 software (BD Biosciences, Franklin Lakes, NJ, USA).

#### 4.3.4. XenoLight DiR Labelling

To detect EV uptake by cells, the undiluted EVs-containing suspension was incubated with 2 µM of XenoLight DiR Fluorescent Dye (PerkinElmer, Waltham, MA, USA) diluted in diluent C (Sigma-Aldrich, Darmstadt, Germany) for 30 min. The resulting solution was then washed twice in sterile PBS. hTCEpi cells were cultured with labelled-HCjE-Gi-derived EVs diluted in conjunctival KSFM (BPE-free), and HCjE-Gi cells were cultured with labelled-hTCEpi-derived EVs diluted in corneal KSFM (BPE-free). As a control, HCjE-Gi cells were cultured with their own unlabeled-derived EVs in conjunctival KSFM (BPE-free) and hTCEpi cells with their own unlabeled-derived EVs in corneal KSFM (BPE-free). This final suspension was analyzed using a BD Accuri C6 flow cytometer (Laser 640 nm, filter 780/60, BD Biosciences, Franklin Lakes, NJ, USA) and the results analyzed using BD Accuri C6 software (BD Biosciences, Franklin Lakes, NJ, USA).

#### 4.3.5. Cellular Uptake of the EVs—DiL Labelling

The collected EVs were labelled with Dil fluorescent dye (V228885, ThermoFisher, Waltham, MA, USA) according to the manufacturer’s instructions. Briefly, EVs were incubated with 0.3% (*V*/*V*) DiI dye in PBS for one hour in the dark at RT followed by two washes in PBS. DiI-labeled EVs were diluted in the respective medium and added to HCjE-Gi or hTCEpi cells for 24 h that were cultured on LabTek chamber slides (ThermoFisher Scientific, Waltham, MA, USA). Following two washes of cells with PBS, the slides were mounted using VECTASHIELD ^®^ mounting medium with DAPI (H-1200, Vector Laboratories, Peterborough, England) and viewed under a Zeiss LSM-700 confocal microscope (BioSciences, Jena, Germany). Control groups were cultured in respective mediums with unlabelled EVs.

### 4.4. Cell Culture with Extracellular Vesicles and Characterisation

#### 4.4.1. Cell Culture with EVs

25,000 cells/cm^2^ were cultured until 80% confluence. KSFM (BPE-free) containing EVs (10.7 × 10^8^ particles/mL) was then added and not refreshed over the course of the experiment. Control cells were cultured with the EVs derived from their own cell type at the same concentration (10.7 × 10^8^ particles/mL).

#### 4.4.2. Characterization Using Real-Time qPCR

The cells were washed at the end of the culture period with sterile PBS and incubated with 350 µL of TRIzol^®^ reagent (Invitrogen, Carlsbad, CA, USA) for 5 min at RT. The solution was collected in an Eppendorf tube. Chloroform (200 µL) (Sigma-Aldrich, Darmstadt, Germany) was added to the tube and the mixture was vortexed for 3–5 s. This solution was then incubated at RT for 15 min and centrifuged at 13,000 rpm for 15 min at 4 °C. The aqueous phase of the solution was collected into a new collection tube and mixed with 500 µL of absolute isopropanol (Sigma-Aldrich, Darmstadt, Germany), which was further incubated for 10 min at RT followed by centrifugation at 13,000 rpm for 10 min at 4 °C. Following the centrifugation step, the resulting RNA pellet was washed with 75% ethanol (Sigma-Aldrich, Darmstadt, Germany) in double-distilled water (ddH_2_O). The resulting solution was centrifuged at 13,000 rpm for 5 min at 4 °C. The pellet was allowed to air-dry for 15 min at RT after discarding the supernatant. The dried pellet was dissolved in 20 µL of DNAse, RNAse-free water (Ambion, Carlsbad, CA, USA). Before reverse transcription assay, the quantity and quality (minimum A260/A280 ratio equal to or greater than 1.8) of RNA was assessed using Nanodrop (ND100, Nanodrop Technologies, Wilmington, DE, USA). 2 µg of RNA template was used for subsequent analysis that were performed in accordance with the Primerdesign’s protocol using Oligo-dT Precision nanoScript *™* Reverse Transcription kit (all reagents were purchased from Primerdesign, Southampton, UK). PCR was performed in a LightCycler 480 II (Roche, Basel, Switzerland) at 95 °C for 2 min for enzyme activation, followed by 45 cycles at 95 °C for 15 s, 60 °C for 60 s 72 °C for 1 s. The melting curves were performed by continuously acquired fluorescence data until the temperature of 95 °C was achieved (at a 0.03 °C/s ramp rate) to further assess the purity of the amplicon. glyceraldehydes-3-phosphate dehydrogenase (GAPDH) was used as a housekeeping gene for each investigated gene. ΔΔCt method was used to calculate the fold increase [71]. Primer sequences are shown in Supplementary Materials Table S3.

#### 4.4.3. Analysis Using Flow Cytometry

The epithelial cells cultured with EVs were trypsinized and centrifuged (all centrifugation steps were performed in RT for 3 min at 1000 rpm) to obtain a single-cell suspension. The resulting cell pellet was re-suspended in 100 µL of 1x FACS Permeabilizing Solution 2 (BD Biosciences, Franklin Lakes, NJ, USA) in ddH_2_O and incubated for 10 min at RT. After centrifugation, the remaining cell pellet was re-suspended in 1 mL of 5% fetal calf serum (FCS) in PBS followed by centrifugation and re-suspension of the pellet in 100 µL of primary antibody (Supplementary Materials Table S4) diluted in PBS and incubated in the dark for 30 min at 4 °C. The resulting cell pellet was washed with 1 mL of 5% FCS in PBS, re-centrifuged and re-suspended in 100 µL of the respective secondary antibody (Supplementary Materials Table S5) diluted in PBS and incubated in the dark for 30 min at 4 °C. The cell suspension was diluted with 1 mL of 5% FCS in PBS and centrifuged followed by re-suspending the cell pellet in 500 µL of 5% (*V*/*V*) FCS in PBS. The final suspension was analyzed using a BD Accuri C6 flow cytometer (BD Biosciences, Franklin Lakes, New Jersey, USA) and the results were analyzed using BD Accuri C6 software (BD Biosciences, Franklin Lakes, NJ, USA).

### 4.5. Exosome Cargo Characterization 

#### 4.5.1. End-Point PCR

Exosomal RNA extraction and cDNA synthesis were performed as mentioned in paragraph 4.4.2. 18 ng of cDNA (corresponding to 2.5μL) was added to a 200μL PCR tube (Appleton Woods, cat. Number BS191) together with REDTaq^®^ ReadyMix ™ (cat. Number R2523, Sigma-Aldrich) (12.5μL), primer pair (5μL) and nuclease-free ddH_2_O (cat. Number AM9937, Ambion) (5μL). HyperLadder 100 bp (cat. Number BIO-33056, Bioline) (5μL) and end-point PCR products (10μL) were loaded into different lanes of a 2% TAE agarose gel (cat. Number A9639, Sigma-Aldrich) supplemented with 0.0036% (V/V) ethidium bromide (cat. Number E1510, Sigma-Aldrich) and allowed to run for approximately 1 h at 100 V (PowerPac Basic ™, BioRad). TAE buffer was prepared using 40 mM Tris (cat. Number 93362, Sigma-Aldrich), 20 mM acetic acid (cat. Number 320099, Sigma-Aldrich), and 1 mM EDTA (cat. Number EDS, Sigma-Aldrich), pH adjusted to 8.6. Gels were scanned using a Chemidoc (chemiDoc ™ XRS+, BioRad) and quantified using ImageLab 5.0 Software.

#### 4.5.2. Exosome Cargo Characterization by Next Generation Sequencing

Total exosomal RNA (1μL) was used to measure small RNA concentration by Agilent Bioanalyzer Small RNA Assay on a Bioanalyzer 2100 Expert instrument (Agilent Technologies, Santa Clara, CA, USA). TailorMix Micro RNA Sample Preparation version 2 protocol (SeqMatic LLC, Fremont, CA, USA) was employed to generate the next-generation sequencing libraries. Briefly, 3′-adapter was ligated to the RNA sample, and excess 3′-adapters were removed subsequently. 5′-adapter was then ligated to the 3′-adapter-ligated samples, followed by first strand cDNA synthesis. Using enrichment PCR, cDNA library was amplified and barcoded. The final RNA library was selected based on the size of 8% TBE polyacrylamide gel. Sequencing was performed on the Illumina NextSeq 500 platform at a read length of 1x75 bp single-end at SR50. FASTQ files for each sample were generated using bcl2fastq software (Illumina Inc., San Diego, CA, USA) and the data were checked using FastQC tool [72] and Bowtie2 to map the spike-in DNA. RNA adapters were trimmed using FastqMcf [73] and cutadapt. PRINSEQ [74] was used in the quality filtering step. Bowtie was used to map against the human reference genome (GRCh37) [73] whereas, the abundance determination and differential expression analysis was performed using DEseq [75].

#### 4.5.3. RNA Isolation and Quantitative Real-Time PCR for miRNA-9-5p

The cells containing EVs pellet were lysed in 450 µL of Trizol, and the small RNA fractions were extracted using mirVana ™ miRNA Isolation Kit (ThermoFisher cat. Number AM1561) according to the manufacturer’s instructions. The purity and quantity of RNA were assessed using NanoDrop (ND1000 Nanodrop Technologies, Wilmington, DE, USA). TaqMAn ™ MicroRNA Reverse Transcription kit was used according to the recommended procedure (ThermoFisher cat. Number 4366596). Reverse transcription reactions were performed on a MasterCycler Gradient 5331 (Eppendorf, Hamburg) with the following conditions: 16 °C for 30 min, 42 °C for 30 min, 85 °C for 5 min, and 4 °C on hold. TaqMan ^®^ Fast Universal PCR Master Mix (2x) no Amperase ^®^ UNG was used for real-time PCR according to the manufacturer’s instructions. Real-time PCR was performed in a QuantStudio 6 Flex Real-Time and PCR System (ThermoFisher) at 95 °C for 10 min, followed by 40 cycles of 95 °C for 10 s and 60 °C for 1 min. miRNA-9-5p expression (ThermoFisher Assay ID 000583, cat. Number 4427975) was analysed against the expression of the small-nucleolar RNA RNU44 (ThermoFisher Assay ID 001094 cat. Number 4427975). The fold increase was calculated using ΔΔCt method [71].

#### 4.5.4. Ex Vivo Study on Human Cadaveric Donor Corneal Tissues—A Pilot Study

**Figure 7:**
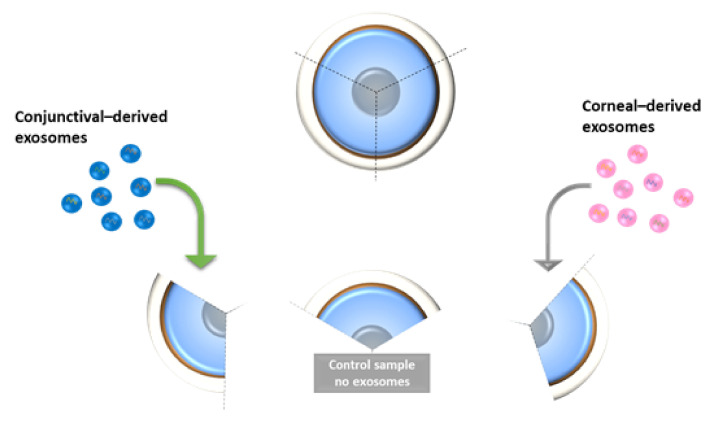
Illustration showing the methodology used for ex vivo study on human cadaveric donor corneal tissues.

Human donor corneo-scleral rims (*n* = 2) were cut into three quadrants using a surgical scalpel (Figure 7). HCjE-Gi and hTCEpi-derived exosomes were extracted from the respective cell lines using ultracentrifugation and mixed in the same media as mentioned above. The tissue pieces were cultured in media containing exosomes and changed daily for up to 7 days. The tissues were placed in Trizol followed by PCR analysis for all the markers as mentioned earlier in addition to HES-1 at day 5 (*n* = 1) and day 7 (*n* = 1).

### 4.6. Statistical Analysis

The Kruskal-Wallis test, followed by a Dunn’s multiple comparison test (unless otherwise specified), and the Mann–Whitney test followed by Bonferroni corrections were used to determine if differences were statistically significant (GraphPad Prism 5, * *p* < 0.05, ** *p* < 0.01, *** *p* < 0.001). Data are expressed as median ± 5–95% percentile (unless otherwise specified). All experiments were repeated in triplicates.

## 5. Conclusions

This is the first study demonstrating that both epithelial cell populations release EVs with exosome-associated features. We have shown that EVs have the ability to influence the transdifferentiation of human conjunctival and corneal epithelial cells, possibly due to differences in the cargo composition between corneal and conjunctival-derived EVs. This process involves a shift in the expression of the corresponding conjunctiva and cornea-associated markers and possibly an intermediate step of cell dedifferentiation. The main differences in cargo composition were related to miRNA molecules with potential roles in cell differentiation processes. The presented data may contribute to a better understanding of the process of corneal conjunctivalisation in pathological conditions. This would further aid in developing therapeutic approaches for the treatment of ocular surface diseases.

## Figures and Tables

**Table 1:** Next-generation sequencing (NGS) assessment of the samples showing miRNA content of the two exosome populations. Mir denotes precursor miRNA molecules, miR denotes mature miRNA molecules.

miRNA	Abundance HCjE-Gi	Abundance hTCEpi	*p*-Value	Log_2_ Fold Change	Role
miR-598	Residual	111.74	0.048	infinity	Downregulated in cancer tissues
miR-34c-3p	46.56	1548.96	0.011	5.1	Upregulated in cell differentiation
mir-34c	116.39	2713.78	0.029	4.5	Upregulated in cell differentiation
mir-146a	10080.83	510.48	0.048	−4.3	Upregulated in stem cell maintenance homeostasis
miR-146a-5p	10080.83	510.48	0.048	−4.3	Upregulated in stem cell maintenance homeostasis
mir-155	703.82	4.38	0.0001	−7.3	Upregulation represses cell differentiation
miR-155-5p	703.82	4.38	0.0001	−7.3	Upregulation represses cell differentiation
miR-9-5p	86.27	Residual	0.03	infinity	Probably involved in cell differentiation
mir-9-1	98.59	Residual	0.018	infinity	Probably involved in cell differentiation
mir-9-2	98.59	Residual	0.018	infinity	Probably involved in cell differentiation
mir-9-3	98.59	Residual	0.018	infinity	Probably involved in cell differentiation

## Data Availability

The data is available at the UCL Institute of Ophthalmology repository. Requests to access the datasets should be directed to Dr. Sajjad Ahmad.

## Supplementary Materials

The following supporting information can be downloaded at: https://www.mdpi.com/article/10.3390/ijms23031718/s1.

## Abbreviations

EC: epithelial cell; EV: extracellular vesicle; KRT: keratin.
