# Supplementary material for: Epithelial Cell-Derived Extracellular Vesicles Trigger the Differentiation of Two Epithelial Cell Lines
Source: Int J Mol Sci. 2022 Feb 2;23(3):1718. doi: 10.3390/ijms23031718 (PMC8836104; doi:10.3390/ijms23031718)
Supplement: Supplementary file 1 [file ijms-23-01718-s001.zip › ijms-1569012-supplementary.pdf]

Antibody	Clone	Host	Isotype	Manufacturer	Dilution
CD63	Polyclonal	Mouse	IgG	Santa Cruz Biotechnology	1:1000
TSG101	4A10	Mouse	IgG1	Abcam	1:1000
GRP94	9G10	Rat	IgG2a	Abcam	1:1000
IgG	---	Mouse	---	ThermoFisher Scientific	0.2μg/μL
IgG1	---	Mouse	---	ThermoFisher Scientific	1μg/μL
IgG2a	---	Rat	---	ThermoFisher Scientific	1μg/μL

Supplementary table S1: List of primary antibodies used for exosome characterization using flow cytometry.

Antibody	Clone	Host	Reactivity	Conjugate	Manufacturer	Dilution
Anti-mouse	A-11005	Goat	Mouse	FITC	Life Technologies	1:1000
Anti-rat	F7512	Sheep	Rabbit	FITC	Sigma-Aldrich	1:1000

Supplementary table S2: List of secondary antibodies used for exosome characterization using flow cytometry.

Primer	Gene Sequence	Product size (bp)
GAPDH	F - #<br>R - #	
KRT3	F – TTCCATCTCAGGCACAAACAA<br>R –CAGGTCCTCCATGTTCTTCAG	130
KRT7	F –CTCCCACCACTCCATCCT<br>R - ATCACTTTCCAGACTGTCTCACT	105
KRT12	F –CTCCAAATCACAAGCACAGTCA<br>R - CCACCTCACCATTCACCATCT	98
KRT13	F –ATGCTGCTGGACATCAAGAC<br>R -TCGTGGTAACAGAGGTGCTA	113
ΔNp63*α*	F –GGAAGGCGGATGAAGATAGC<br>R - CATGTGTGTTCTGACGAAACG	96
ABCB5	F -GGAAAGAATTACCACTACCAAGAAGG<br>R - TGGTAGCATCCAAATGGGCAAAC	119

Supplementary table S3. List of primers used for Real Time qPCR studies. The primer sequences and the product size are included. Abbreviations used: GAPDH: glyceraldehyde-3-phosphate dehydrogenase, KRT: keratin, ABCB5: ATP binding cassette sub-family B member 5. # Sequences and product size of reference genes are not commercially provided by PrimerDesign.

Antibody	Clone	Host	Isotype	Manufacturer	Dilution
KRT3	AE5	Mouse	IgG	Abcam	1:1000
KRT7	RCK105	Mouse	IgG1	Santa Cruz	1:500
KRT12	J6	Rabbit	IgG	Santa Cruz	1:1000
KRT13	Ks13.1	Mouse	IgG1	Santa Cruz	1:500
ΔNp63α	Poly6190	Rabbit	IgG	BioLegend	1:1000
IgG	---	Mouse	---	ThermoFisher Scientific	#
IgG	---	Rabbit	---	ThermoFisher Scientific	#
IgG1	---	Mouse	---	ThermoFisher Scientific	#

Supplementary table S4. List of primary antibodies used for flow cytometry. # Concentration dependent on primary antibody. Abbreviations used KRT: keratin

Antibody	Clone	Host	Reactivity	Conjugate	Manufacturer	Dilution
Anti-mouse	A-11005	Goat	Mouse	FITC	Life Technologies	1:1000
Anti-rabbit	F7512	Sheep	Rabbit	FITC	Sigma-Aldrich	1:1000

Supplementary table S5. List of secondary antibodies used for flow cytometry. Abbreviations used FITC: fluorescein isothiocyanate
